# Around the Hospitals

**Published:** 1894-07-28

**Authors:** 


### Around the Hospitals.

[Notice to the Managers of Institutions.—Special space is now reserved for the insertion of notices of hospital meetings and festivals. The Editor requests that all notices may reach The Hospital Office, 428, Strand, at least one week before the date of each meeting.]

**The London Fever Hospital.**—This institution fulfils a most necessary and useful part in the scheme of medical relief of the metropolis. It has had very hard work for two years, being nearly the whole time continuously full owing to the unusual prevalence of scarlet fever. In 1892 it was thought that the highest record had been reached, but the events of 1893 showed that this was not so, and a yet greater number of patients were received. It is greatly to the credit of the institution that the death-rate from so grave a disease as scarlet fever should only reach 2·3. During the same period the death-rate from this disease in the rest of London was 4·3 per cent. Measles found its way into the hospital, in spite of all due care exercised, and twelve patients were attacked. Of the five cases of diphtheria which were treated one died. The institution also lost a valued medical officer, Dr. Ellis Durham, through this disease. The two years of heavy work which have so taxed the resources of the hospital have brought into special prominence the need of an auxiliary institution in the shape of a convalescent home. This it is hoped charitable friends will assist in securing. The hospital itself cannot contemplate such an expenditure at present, having to attend to the alterations and additions necessary for the parent building. The drains are now set in order, and a most complete home has been erected for the nurses. These two improvements have been effected at a cost of £4,985. The ordinary income in 1893 amounted to £13,366, and the total receipts to £18,965. The ordinary expenditure was £9,802, and the total expenditure £14,945. All the legacies were invested, and a sum placed at the bank to defray the costs of the month of December, 1893, up to which date the accounts were rendered. It will be seen that the hospital is in a flourishing condition, and is therefore able to offer an exceedingly liberal return for those who render themselves governors of the institution. During the past year 893 patients were treated.

